# Characterisation of sugar beet (*Beta vulgaris *L. ssp. vulgaris) varieties using microsatellite markers

**DOI:** 10.1186/1471-2156-11-41

**Published:** 2010-05-18

**Authors:** Marinus JM Smulders, G Danny Esselink, Isabelle Everaert, Jan De Riek, Ben Vosman

**Affiliations:** 1 Plant Research International, Wageningen UR Plant Breeding, PO Box 16, NL-6700 AA Wageningen, The Netherlands; 2 Unit Plant, Institute for Agricultural and Fisheries Research ILVO, Caritasstraat 21, B-9090 Melle, België

## Abstract

**Background:**

Sugar beet is an obligate outcrossing species. Varieties consist of mixtures of plants from various parental combinations. As the number of informative morphological characteristics is limited, this leads to some problems in variety registration research.

**Results:**

We have developed 25 new microsatellite markers for sugar beet. A selection of 12 markers with high quality patterns was used to characterise 40 diploid and triploid varieties. For each variety 30 individual plants were genotyped. The markers amplified 3-21 different alleles. Varieties had up to 7 different alleles at one marker locus. All varieties could be distinguished. For the diploid varieties, the expected heterozygosity ranged from 0.458 to 0.744. The average inbreeding coefficient F_is _was 0.282 ± 0.124, but it varied widely among marker loci, from F_is _= +0.876 (heterozygote deficiency) to F_is _= -0.350 (excess of heterozygotes). The genetic differentiation among diploid varieties was relatively constant among markers (F_st _= 0.232 ± 0.027). Among triploid varieties the genetic differentiation was much lower (F_st _= 0.100 ± 0.010). The overall genetic differentiation between diploid and triploid varieties was F_st _= 0.133 across all loci. Part of this differentiation may coincide with the differentiation among breeders' gene pools, which was F_st _= 0.063.

**Conclusions:**

Based on a combination of scores for individual plants all varieties can be distinguished using the 12 markers developed here. The markers may also be used for mapping and in molecular breeding. In addition, they may be employed in studying gene flow from crop to wild populations.

## Background

Sugar beet (*Beta vulgaris *L.) is a crop of major importance for sugar production in temperate zones. Varieties are produced through crosses of diploid male sterile (CMS) lines with tetraploid, or increasingly, diploid pollinator lines, resulting in triploid or diploid varieties, respectively [1]. As the parental lines are mixtures of genotypes, the varieties will consist of mixtures of plants from various parental combinations. This leads to some problems in variety registration research. Variety registration is based on Distinctiveness, Uniformity, and Stability (DUS) research. Using a visual inspection of morphological characteristics, distinctiveness from other varieties is not easy to assess, for several reasons: the crop has a narrow genetic basis [2,3], which results in varieties that are highly similar in appearance [4], the varieties are mixtures of genotypes, and breeders change the pollinator line in modern hybrids frequently to produce locally adapted hybrid varieties. For these reasons, the other two aspects of the standard DUS research, uniformity and stability, are not determined, and there are no UPOV (International Union for the Protection of New Varieties of Plants) guidelines for this crop.

The number of informative morphological characteristics is limited. Therefore, most often production-related characteristics as beet yield, sugar content and total sugar yield are included as descriptors. A preliminary characterisation ("pre-screening") of newly submitted varieties with molecular markers during the winter before sowing could be of help in the planning of the field trials and may give a first indication for distinctiveness, provided that a sufficient number of markers is used and that overall marker profile and phenotype correlate well.

Molecular markers have been used successfully for variety identification in a large number of crops, including selfing species [5,6] and clonally propagated plants [7,8]. In sugar beet, RFLP, RAPD, and AFLP [9-15] studies have been reported. Although AFLP markers are reproducible between laboratories [16,17], data base building can be a problem as different equipment may lead to different profiles. Six co-dominant microsatellites were used to study genetic diversity in wild, cultivated, and weedy forms of *Beta vulgaris *[18,19]. Rae et al. [20] developed a set of mostly dinucleotide repeats for incorporation into the linkage map of *B. vulgaris*, and Richards et al. [21] characterized eight new polymorphic microsatellite markers, of which five were based on trinucleotide repeats. Cureton et al. [22] developed six microsatellite markers to measure gene flow in sea beet (*Beta vulgaris *ssp. maritima). Laurent et al. [23] mapped a large number of genomic and EST-derived microsatellites on a genetic map of sugar beet, the majority of which were dinucleotide repeat markers.

To be useful for identification of varieties the markers should allow determining unequivocally the genotype of each plant independently. The ease and accuracy of scoring varies among microsatellite markers, with significantly more problems when applying dinucleotide repeats, due to their tendency to generate more stutter bands, which may co-migrate with neighbouring alleles. The experience in those species in which large replication studies have been set up among laboratories, is that rigorous screening of markers is necessary [5,6]. For that reason we have developed a set of new microsatellite markers for *B. vulgaris *with PIG-tailed reverse primers [24] and stringent quality demands (Quality 1 or 2 of Smulders et al. [25]). We have applied this set of 12 di-, tri- and tetranucleotide repeat microsatellite markers to determine the genetic variation within and between 40 diploid and triploid varieties. Using 30 plants per variety we have generated a dataset of genotypes of 1200 plants. We analysed the data with respect to allelic diversity, and discuss applications of the markers in sugar beet, sea beets, and ruderal beets.

## Results

### Microsatellite marker development

For accurate genotyping of varieties and database building, high quality microsatellite markers are needed. Therefore the isolation of microsatellites was focussed on tri- and tetranucleotide repeats, although dinucleotide repeats were isolated as well. In total 3200 clones were screened for microsatellite-containing inserts. In total 31 clones (1%) were found positive for tetranucleotide motives, 240 (7.7%) for trinucleotide repeats and 240 (7.7%) for dinucleotide repeats. For 65 unique microsatellite sequences, primer pairs were designed on the flanking regions. For each locus the amplification pattern was evaluated with respect to pattern quality and degree of polymorphism on a set of individual plants of 10 varieties originating from different breeders. Twenty-five primer pairs (39%) produced polymorphic and simple banding patterns. These primers were selected for further analysis with fluorescent primers on an ABI 3700 using the same set of test varieties. The twelve most robust markers showing no or moderate stutter bands, a low degree of differential amplification, and easy scorability, were used for genotyping the sugar beet varieties (Table 1). These 12 markers consisted of two perfect and four compound dinucleotide repeat loci, five trinucleotide loci, and one locus with both a perfect dinucleotide repeat and a perfect tetranucleotide repeat.

**Table 1:** Characteristics of 25 newly developed sugar beet microsatellite markers.

Micro-satellite marker^1^	EMBL Accession number	Forward primer (5'-3') Reverse primer^2 ^(5'-3')	Repeat motif^3^	Annealing Temp	Predicted size	Quality^4^
Bvv01	[EMBL:AM410749]	CCATATGGAGGGGTAGAGCA	(GGA)_4-1_(GGT)_7_	55	105	1
		GTTTGCACCATAGGCACCACCACTTG				
Bvv10	[EMBL:AM410750]	CTTTGAGAATTGAGATACTATG	(CA)_56-3_	52	212	1
		GTTTGTCTGGACGCAAGCACAC				
**Bvv15^5^**	[EMBL:AM410751]	TGCTGACCTTGCAGTTAATAAGTT	(CAA)_34-7_	54	298	1
		GTTTCATGTGATGGCTTGCTTTCTAA				
**Bvv17**	[EMBL:AM410752]	CGACGCCTTTTTGAAGGAATAGGAT	(GAT)_12-3_	57	128	1
		GTTTCACCCCTGGGTCCTGATCTACAAC				
Bvv18^6^	[EMBL:AM410753]	CACCATAACCGCCCCCACCATAAT	(GCC)_3_(ACC)_3_	60	208	1
		GTTTCTTGGCCGTAGGGTAAGGGTCAACTA				
**Bvv21**	[EMBL:AM410754]	TTGGAGTCGAAGTAGTAGTGTTAT	(GGC)_13-5_	53	250	1
		GTTTATTCAGGGGTGGTGTTTG				
Bvv22	[EMBL:AM410755]	CTATGCATCGCCCAATAATTACTTAA	(CCG)_5_(CCA)_5_	52	210	1
		GTTTATATAACACTGCTTATTTAATGTCC				
**Bvv23**	[EMBL:AM410756]	TCAACCCAGGACTATCACG	(GA)_16_	50	109	1
		GTTTACTGACAAAGCAAATGACCTACTA				
Bvv25^7^	[EMBL:AM410757]	GAAACCACATAAAAACCCCTCTTA	(TCA)_10_	51	121	2
		GTTTCAAGTAGTCCCGTTAACATCTGA				
Bvv27	[EMBL:AM410758]	GGGTTCATCATCATCCTTATCATT	(TCA)_13-3_, (TCA)_35-10_	54	310	1
		GTTTACGCTCCTCCATCATCAGACCA				
**Bvv30**	[EMBL:AM410759]	TGTGCCCAAAATCCTGAA	(GA)_31_	51	183	1
		GTTTAATTGGCTGGGTAAAAGAGA				
Bvv31	[EMBL:AM410760]	AGAAGCCTTTAAAATCCAACT	(CA)_14_, (GTAT)_69_	49	461	2
		GTTTACAGCGTCTCACCATAAGT				
**Bvv32**	[EMBL:AM410760]	AGAAGCCTTTAAAATCCAACT	(CA)_14_	50	142	2
		GTTTACATATGGAACTTTAATGAACAAGTGATAT				
Bvv37	[EMBL:AM410761]	TGGACGCCATATTAGAAGAT	(GT)_27_	50	216	1
		GTTTATACAAATGAATATGAGAATACTG				
**Bvv43**	[EMBL:AM410762]	TGACACTCTTCTTTGCAACACATAA	(GT)_96-18_	54	257	2
		GTTTGTAAATGTTGCAAAATATTGGTAT				
Bvv45	[EMBL:AM410763]	GTATAGCAAAAGTCATTTTGTTTGTGT	(GT)_14-1_, (CGCA)_8_	55	230	1
		GTTTCTCGGCCTTCCCTTTCTAATGTCTAG				
Bvv48	[EMBL:AM410764]	GGCTTCCCTAGACAACC	(GT)_24_	50	209	1
		GTTTATAGGCAAATGAATGAGG				
**Bvv51**	[EMBL:AM410765]	AGCAAAACTTATCTCAAATCTGG	(TG)_9_(AG)_32-1_	51	272	1
		GTTTGTCTACCGTGGCTGTGC				
**Bvv53**	[EMBL:AM410766]	CATGTCGAGGAGTGAGTTCAGGAA	(GT)_17_, (GA)_35_	53	226	1
		GTTTCAACTATAGGTGCATCTTTTAC				
Bvv54	[EMBL:AM410767]	ATCTGCATGCCGTCACTC	(TC)_12_(AC)_12_	52	279	1
		GTTTCACTGTACCTTCGAATGTTAG				
Bvv57	[EMBL:AM410768]	CATTACCATGGGAACGAA	(GT)_25_	50	232	1
		GTTTAAGGGATACAATGTTAGTTATGAA				
**Bvv60**	[EMBL:AM410769]	AAGAATGCTTCAACTTTTTCATGG	(CAT)_7_, (CAT)_7_, (CAT)_11-1_	52	256	1
		GTTTAGGGTCGGATATAAGAGGGAGTGG				
**Bvv61**	[EMBL:AM410770]	ATGGGAGAATATTGGTGACA	(GAA)_22_	49	200	1
		GTTTGCCACAAATCATCTCTACTAA				
Bvv62	[EMBL:AM410771]	ATGGCAATGCGCAGAATAACC	(CAG)_11-2_	54	155	1
		GTTTGCTGAGGAGGCTGCATTTGTT				
**Bvv64**	[EMBL:AM410772]	TTTTTGGGAGTTTCATCACTACTTT	(CT)_18-1_(CA)_19_	51	207	2
		GTTTCATATAAGGGGAGTCTTCTCACAA				

^1 ^In bold the 12 markers that have been used to genotype 40 cultivars (1200 plants) (see Table 2). They were amplified separately but combined before analysis on an ABI sequencer, as follows: multiplex 1 consisted of markers Bvv15, Bvv30, and Bvv64; multiplex 2 of Bvv17, Bvv43, and Bvv61; multiplex 3 of Bvv 51, Bvv 53, and Bvv60; multiplex 4 of Bvv 21, Bvv23, and Bvv32

^2 ^GTTT is a pigtail [24]

^3 ^the number after the minus sign is the number of imperfect repeats. For instance, (CA)_56-3 _means that the microsatellite repeat covers of length of 56 (CA) repeat units, but of these 3 are not (CA).

^4 ^according to Smulders et al. [25]

^5 ^The sequence of the forward primer of Bvv15 was found in cDNA clone EO12340

^6 ^The sequence of the cloned Bvv18 fragment was found in cDNA clone EG551697

^7 ^The sequence of the cloned Bvv25 fragment was found in BAC clone ED032383

### Alleles detected

For the evaluation of the markers 30 individual plants per variety were genotyped. Table 2 shows the number of alleles detected for each marker, which varied widely (from 3 to 21), but the effective number of alleles was quite comparable across loci (1.95-3.74; Table 2). In total 91 different alleles were detected. From the number of dropouts in amplification and the positive Fis values we deduced that null alleles may exist. Additional population-genetic parameters of these varieties are listed in Additional file 1.

**Table 2:** The number of microsatellite alleles detected in 30 individual plants per variety.

			Common, rare alleles found using microsatellite marker												
				
Variety	Seed Company	Ploidy level	Bvv15	Bvv17	Bvv21	Bvv23	Bvv30	Bvv32	Bvv43	Bvv51	Bvv53	Bvv60	Bvv61	Bvv64	Total number of alleles
A8106	Agrosem	3	5,1	2,0	2,0	3,0	3,0	3,0	5,2	4,0	4,0	2,0	4,1	5,0	42
Ariana	KWS	3	4,1	2,0	3,0	2,0	2,0	3,1	5,0	4,1	3,1	3,0	6,1	5,0	42
Aristo	Novartis	2	4,2	2,0	2,0	4,0	3,1	2,0	2,0	4,0	2,0	2,0	6,1	4,0	37
Assist	SES	3	5,2	2,0	3,0	4,0	3,0	3,0	3,0	4,0	4,2	2,0	5,1	4,1	42
Atlantis	Van der Have	3	6,0	2,0	1,0	4,1	3,0	2,0	2,0	5,1	5,0	2,0	4,2	6,0	42
Aurelia	KWS	3	3,0	1,0	1,0	4,1	2,0	2,0	3,0	4,0	2,0	3,0	3,1	6,2	34
Blenheim	Van der Have	3	4,1	2,0	2,0	3,1	3,0	3,0	3,2	5,0	4,0	2,0	5,0	3,1	39
Brigitta	KWS	2	3,0	2,0	2,0	1,0	2,0	1,0	3,0	4,0	4,0	2,0	4,2	2,0	30
Caramel	Kuhn	3	5,0	2,0	1,0	4,0	2,0	2,0	2,0	4,0	4,0	2,0	2,0	3,0	33
Claudia	CFS	3	4,0	2,0	2,0	3,0	3,1	3,1	5,0	5,0	6,3	2,0	6,3	5,2	46
Crestor	Novartis	2	3,0	2,0	3,0	2,0	2,0	2,0	2,0	4,0	2,0	2,0	5,1	3,1	32
Cynthia	KWS	3	4,1	2,0	2,0	4,0	2,0	3,0	3,0	5,0	4,0	2,0	3,0	3,0	37
DS3014	Danisco	3	3,0	2,0	2,0	2,0	2,0	2,0	1,0	4,0	2,0	2,0	6,3	2,0	30
DS3030	Danisco	3	4,0	2,0	2,0	3,1	2,0	3,0	3,0	3,0	4,1	2,0	5,1	3,0	36
Fortis	Hilleshog	2	2,0	2,0	2,0	3,0	3,0	3,0	2,0	4,1	2,0	2,0	4,1	6,0	35
H66377	Van der Have	3	5,2	2,0	2,0	4,1	3,1	3,0	4,2	4,1	3,0	2,0	5,2	6,2	43
H66411	Van der Have	3	7,0	2,0	2,0	4,0	2,0	3,0	3,1	5,0	4,0	2,0	3,0	5,1	42
Helsinki	Van der Have	3	6,1	2,0	2,0	3,0	3,0	3,1	1,0	5,0	4,0	2,0	4,1	3,0	38
HI0032	Novartis	2	3,1	2,0	2,0	3,0	3,1	3,0	4,0	3,0	4,1	2,0	4,0	5,0	38
HM5432	Hilleshog	3	6,1	2,0	2,0	3,0	2,0	3,0	4,0	4,0	3,0	3,0	3,1	5,1	40
KWS8123	KWS	2	3,1	2,0	2,0	2,0	2,0	2,1	1,0	3,0	1,0	2,0	2,1	5,1	27
KWS9226	KWS	3	5,0	1,0	1,0	2,0	2,0	2,0	4,0	3,0	3,0	3,1	4,1	4,1	34
Lenora	KWS	2	4,2	2,1	2,0	2,0	2,0	1,0	1,0	3,0	3,0	2,0	3,0	1,0	26
Lion9909	Lion Seeds	3	5,0	2,0	2,0	4,1	2,0	3,0	3,0	4,1	5,1	2,0	6,0	4,1	42
Lion9912	Lion Seeds	3	6,0	2,0	2,0	3,0	2,0	3,0	2,0	4,0	5,0	2,0	5,1	3,1	39
Madonna	KWS	2	3,1	1,0	2,0	2,0	2,0	2,0	2,0	4,0	3,0	2,0	4,0	2,0	29
MK9907	Kuhn	3	4,0	2,1	2,0	2,0	3,0	3,0	3,1	5,2	4,0	3,0	4,0	4,0	39
Nemil	Novartis	2	6,2	2,0	3,0	3,0	2,0	3,0	1,0	5,2	2,0	2,0	6,2	3,1	38
Opus	Dickman	3	7,0	2,0	2,0	4,0	3,0	3,0	3,0	4,0	5,0	2,0	5,1	5,2	45
Oslo	Van der Have	3	5,0	2,0	2,0	4,0	3,0	2,0	3,0	4,0	4,0	2,0	5,0	3,0	39
Princesse	Delitzsch	3	3,1	2,0	2,0	4,0	2,0	2,0	4,0	5,0	3,0	2,0	5,1	4,1	38
Ravel	Kuhn	3	4,0	2,0	2,0	4,1	3,0	2,0	1,0	5,0	4,0	2,0	4,1	4,0	37
Rebecca	KWS	2	4,2	2,0	2,0	3,0	2,0	3,0	3,0	5,1	5,2	2,0	3,0	4,0	38
S1901	SES	3	5,0	2,0	2,0	4,0	2,0	2,0	2,1	4,0	4,0	2,0	4,1	4,1	37
Stru2001	Fr Strube Saatzucht	2	3,0	2,0	1,0	1,0	1,0	2,0	1,0	3,0	3,0	2,0	4,1	2,0	25
Sylvester	Van der Have	3	7,2	2,0	2,0	3,0	3,0	3,0	2,1	4,0	3,0	2,0	5,2	5,0	41
Tiara	KWS	3	4,0	1,0	2,0	3,1	3,0	2,0	3,0	4,0	5,0	2,0	4,0	3,0	36
Toledo	Novartis	3	4,0	2,0	2,0	4,0	2,0	2,0	3,1	4,0	4,1	2,0	6,0	3,0	38
Winner	Kuhn	3	6,0	2,0	1,0	4,0	3,0	2,0	2,0	4,0	4,0	2,0	4,1	5,1	39
Winsor	Novartis	3	4,0	2,0	2,0	3,0	2,0	3,0	4,1	5,0	4,0	2,0	6,0	4,0	41
Allele length range			201-293	135-138	236-268	092-108	136-143	133-141	257-288	240-281	174-234	252-275	213-370	224-237	
Total number of alleles (in 1200 plants)			11	3	5	5	4	4	9	8	10	6	21	6	92
Effective number of alleles (in 1200 plants)			2.7	3.0	2.2	3.5	2.2	3.2	2.6	3.7	3.1	2.0	3.1	3.1	
He (expected heterozygosity) (in 316 diploid individuals)			0.525	0.626	0.529	0.686	0.543	0.672	0.700	0.645	0.589	0.458	0.659	0.744	

### Variety characterization based on dominant scoring of alleles

Using the set of 12 marker loci, we found 25-38 different alleles (on average 32.3 per variety) in the 30 plants of a diploid variety and 33-46 (average 39.0) alleles in a triploid variety (Table 2). In general, individual plants from varieties reported to be diploid had only one or two alleles per locus. There were only 15 out of 330 plants from reportedly diploid varieties with three different alleles at one or two loci (5 plants each of Rebecca and Brigitta, 3 of Nemil, 1 each of HI0032 and Fortis). On average, diploid plants had 1.3 alleles per locus. Among plants of the triploid varieties, the average number of alleles per locus was 1.6. Depending on the locus, between 0 (markers bvv17 and bvv21) and 183 (bvv15) plants contained three different alleles at a single locus. Overall, 528 of the 870 plants of these varieties had three different alleles at one or more marker loci, underlining a considerable amount of genetic variation present within these plants.

Triploid varieties are produced from tetraploid males and diploid female plants. While females are always diploid and may be shared between diploid and triploid varieties, the male plants are either diploid or tetraploid and these may form genetically distinct groups. However, tetraploid lines can also easily be made from diploids. When we calculated genetic differentiation between diploid and triploid groups (330 and 870 plants, respectively) F_st _= 0.1327 across all loci, ranging from 0.037 for marker bvv30 to 0.2066 for bvv23. Each of these estimates was highly significant (p < 0.001, tested by permutation of individual plants among all varieties).

This differentiation between diploid and triploid varieties could also be the result of the fact that some breeders specialise in diploid varieties, and others in triploids. If so, it would reflect differentiation among breeders rather than between ploidy levels. We therefore also tested the differentiation among breeding companies. Among breeders, we found F_st _= 0.0628 ± 0.0092, which is roughly half of the difference between diploid and triploid varieties.

### Genetic diversity and differentiation among varieties

**Figure 1:**
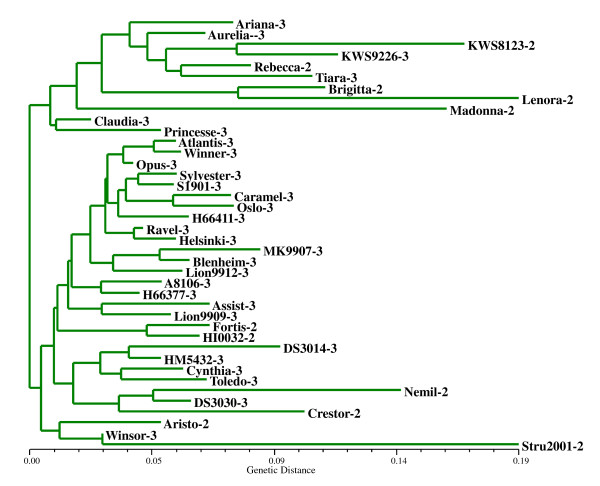
**Neighbour-joining tree based on pairwise genetic distances between sugar beet varieties**. The genetic distances were calculated using dominant scoring of alleles. The names of the varieties are followed by their ploidy level: 2 = diploid (2n = 2x), 3 = triploid (2n = 3x).

A NJ tree was made using the pairwise genetic distances between varieties to visualise the genetic distances among varieties (Figure 1). It shows that the genetic distance is, on average, larger among diploid varieties. For instance, the inner part of the dendrogram contains 17 triploid varieties at relatively small distances from each other. The same pattern is visible in a PCO plot, with the triploid varieties central in the plot and the diploid varieties further from each other (Additional file 2). Triploid varieties have a higher probability of sharing alleles due to the fact that they have more gene copies, hence on average more alleles, which may explain the pattern observed.

There is no clear structure in the genetic relatedness of varieties from particular breeding companies in the tree, except that the top branch consists exclusively of nine varieties from KWS (Ariana, Aurelia, KWS8123, KWS9226, Rebecca, Tiara, Brigitta, Lenora, and Madonna).

Overall, F_st _= 0.133, but this value was lower among triploid varieties (F_st _= 0.100) and much higher among diploid varieties (F_st _= 0.232) (Table 3), which is consistent with the pattern observed in the dendrogram. The correlation between the values of individual marker loci for triploid and diploid varieties is relatively poor (R^2 ^= 0.54), suggesting that the gene pool differences between triploid and diploid varieties are not evenly spread across loci.

**Table 3:** F-statistics of 40 varieties genotyped with 12 microsatellite markers.

	Dominant scoring			Co-dominant scoring					
			
	all	triploids	diploids		diploids				
			
	Fst	Fst	Fst	Fst	Fis	Fit	Ho	Hs	Ht
			
varieties	40	29	11	11	11	11	11	11	11
plants	1200	870	316	316	316	316	316	316	316
bvv15	0,095	0,079	0,140	0,135	-0,113	0,038	0.513	0.460	0.525
bvv17	0,158	0,127	0,257	0,291	0,729	0,808	0.122	0.453	0.623
bvv21	0,181	0,168	0,239	0,257	0,324	0,498	0.272	0.397	0.525
bvv23	0,207	0,145	0,421	0,459	0,820	0,903	0.069	0.383	0.683
bvv30	0,037	0,015	0,119	0,204	-0,011	0,196	0.445	0.442	0.543
bvv32	0,172	0,129	0,288	0,345	0,408	0,613	0.271	0.455	0.670
bvv43	0,168	0,107	0,279	0,291	0,876	0,912	0.065	0.516	0.703
bvv51	0,095	0,082	0,152	0,180	-0,152	0,055	0.615	0.533	0.641
bvv53	0,132	0,106	0,208	0,274	-0,232	0,106	0.542	0.442	0.587
bvv60	0,047	0,034	0,093	0,151	-0,355	-0,150	0.533	0.394	0.457
bvv61	0,119	0,094	0,195	0,198	0,433	0,545	0.260	0.527	0.658
bvv64	0,151	0,096	0,299	0,349	0,582	0,728	0.206	0.504	0.744
Jackknifed estimators (over loci)							Overall		
Mean	0,133	0,100	0,232	0,271	0,282	0,479	0.326	0.459	0.613
SE	0,014	0,011	0,027	0,028	0,124	0,105			

The estimate of F_is _for the whole dataset was negative for each of the markers (not shown), which is most likely an artefact of the dominant scoring of the markers. In theory, this can influence the F_st _estimates as well. For the diploid varieties we were able to estimate the magnitude of this effect through a comparison with an analysis using codominant scoring (assuming two alleles per locus per plant and no null alleles). Table 3 (middle panel) shows that the actual F_is _value varies widely among marker loci, from F_is _= +0.876 (heterozygote deficiency) to F_is _= -0.350 (excess of heterozygotes), with an average of F_is _= 0.282 ± 0.124, Table 3). The effect on the estimation of the variation present among varieties (F_st_) is limited: F_st _averaged across loci is 0.232 for dominant scores (left panel) and 0.271 for codominant scores (middle panel; 17% more). The F_st _estimates for most loci are close to this systematic difference of 17%, and the pairwise correlation between the values per locus is R^2 ^= 0.91. This indicates that differentiation among diploid varieties is being estimated comparably using dominant or codominant scores.

## Discussion

We have developed a set of new microsatellite loci for sugar beet, which amplified 2-21 alleles per locus. This is comparable to the 2-11 alleles found by Richards et al. [21] for their microsatellite markers in a set of sugar beet and sea beet plants. Desplanque et al. [18] and Viard et al. [19] found up to 10 alleles for a marker in a single variety. This level of gene diversity does not seem to correspond with the notion of little genetic variation in the crop sugar beet due to a bottleneck during its development from wild beets [1]. The breeding system, which employs separate gene pools for paternal and maternal parents, increases the gene diversity within individual plants, and the habit of working with pools of parental plants, which contain a large amount of genetic diversity [19], may contribute to the fact that 84-92% [26] of the genetic variation of the crop is present within hybrid varieties.

### Ploidy level

We have applied 12 of our markers to analyse 30 plants of each of 40 sugar beet varieties.

The markers detected only few (15/330) triploid plants in diploid varieties. The highest frequencies of triploid plants were found for two varieties (5/30 plants each for Brigitta and Rebecca). These plants are probably the result of pollination by tetraploid pollen donors from production fields for other, triploid, varieties in the neighbourhood of the seed production fields of the diploid varieties. In Europe seed production of sugar beet varieties takes place in the South-West and South-East of France, Northern Italy, and the South of Ukraine, and in these areas the distance between production fields is at least 1000 m to severely limit cross-pollination, but this cannot be avoided completely. Accidental cross-fertilization may also take place with ruderal populations in the vicinity of the seed production fields [27,28], but this would produce diploid offspring.

### Genetic differentiation

The overall genetic differentiation between diploid and triploid varieties was F_st _= 0.1327 across all loci. Part of this differentiation coincides with the differentiation among breeders' gene pools, which was F_st _= 0.0628. This suggests that breeders use parental lines that are, to some extent, genetically different. The latter value can be expected to gradually decrease in the future, as there have been mergers between sugar beet breeding companies in recent years, which may result in merging of the breeding programs.

When partitioning the genetic variation using F statistics, the estimate of F_is _of diploid plants turned out to be highly variable among microsatellite loci: from F_is _= 0.876 (large shortage of heterozygotes) to F_is _= -0.35 (excess of heterozygotes). The excess of heterozygotes is not surprising as the propagation system pairs selected male-sterile (CMS) mother lines with selected father lines, with the aim of assortative mating and hybrid seed production. The shortage of heterozygotes at some marker loci may indicate selection. It may also indicate the presence of null-alleles, i.e. alleles that have gone undetected, or skewed inheritance [12]. Laurent et al. [23] found 14% skewed segregation in an F2 population, notably for markers on linkage group V [29]. Viard et al. [19,30] found significant heterozygote deficiencies in weed beets. Fénart et al. [1] observed also significant deviations in F_is_, in both directions, in wild sea beet and weed beet populations. Viard et al. [19] thought it may be related to a low frequency of self-compatibility alleles commonly used in breeding programs. This was recently confirmed by Arnaud et al. [27].

Nonetheless, F_st _values of dominantly scored and codominantly scored markers (for diploid varieties) were in good concordance, indicating that regardless of the statistical analysis of the data, genetically similar and dissimilar varieties can be distinguished reliably. This is in agreement with the conclusions of De Riek et al. [24], who compared the power of these microsatellites with that of a set of AFLP markers. The differentiation among diploid varieties was quite high: F_st _ranged from 0.093 to 0.421 (Table 3). The average of 0.232 is higher than Fénart et al. [1]'s estimate of F_st _= 0.082 among 13 diploid sugar beet varieties using 5 microsatellite markers, which in turn was higher than the differentiation among weed beets and among sea beets. It would be interesting to determine the level of differentiation assessed with our markers among these groups of beets.

### Applications

Based on a combination of scores for individual plants all varieties can be distinguished using the 12 markers employed here. However, as the varieties are mixtures of genotypes, not all individual plants can always be identified or classified unequivocally. De Riek et al. [26] compared various ways of analysing the data for eight of these varieties. They concluded that, using the data for 30 individual plants for each variety, assignment methods accomplished a very good distinction among the genetically diverse varieties. In their assignment-based method, for each individual plant the 10 most genetically similar partner plants were identified across the whole data set. The origin of these highest-ranking plants was then used to assign the plants to a particular variety. With microsatellite data, between 24 and 30 of the 30 plants analysed for each variety, were assigned correctly to this variety. The partitioning of the origin of the highest-ranking partners over all varieties in the dataset was also used to develop an assignment-based similarity measure for such sets of mixtures of genotypes, called similarity-by-assignment (Sa_x, y_) [26].

## Conclusions

Microsatellite markers may be used for genetic mapping and breeding purposes [29]. The markers developed here were polymorphic within all or nearly all varieties, which indicates that they may be used for mapping in most crosses in sugar beet. In addition, they may be employed in studies of crop-to-wild gene flow [1], including those in the frame of biosafety studies [31].

## Methods

### Plant material

For the isolation of microsatellites, genomic DNA of *Beta vulgaris *L. *ssp. vulgaris *variety Holly was used. For the characterization of varieties, 30 individual plants of 40 varieties (listed in Table 2) were analyzed (in total 1200 plants). Young leaves of a single individual were harvested, immediately frozen in liquid nitrogen and stored at -80°C until use.

### DNA extraction

For the construction of a genomic library enriched for microsatellites, nuclear DNA of high quality was extracted from leaves of variety Holly according to Vosman et al. [32]. For microsatellite amplification, DNA of single individuals was extracted from freeze-dried leaves either according to Fulton et al. [33] or by a combination of this method with the Qiagen Dneasy Plant Mini kit (Westburg, The Netherlands). In the combination extraction protocol, after chloroform extraction the cleared supernatant was mixed with Qiagen binding buffer (AP3/EtOH) and applied to a DNeasy spin column (Esselink, unpublished). Subsequently, the column was washed and DNA eluted. Typical yield of this extraction protocol was 20 μg DNA per 20 mg dried weight.

### Microsatellite isolation

Microsatellites were isolated from enriched small-insert genomic libraries essentially as described by Van de Wiel et al. [34] and Esselink et al. [7]. The DNA was digested with Alu I, Mbo I or Rsa I, and the enrichment was carried out using filter-immobilized synthetic dinucleotide [(GT)_12_, (GA)_12_], trinucleotide [(TCT)_10_, (TGT)_9_, (GAG)_8_, (GTG)_8_, (TGA)_9_, (AGT)_10_, (CTG)_8_, (CGT)_8_], and tetranucleotide [(GATA)_8_, (TGTT)_8_, (GTAT)_8_] repeats, all in separate reactions. Primers were designed on the obtained sequences using primer3 http://primer3.sourceforge.net/.

### Microsatellite amplification and detection

Microsatellites were amplified in a 20 μl reaction volume containing 20 ng of genomic DNA, 2-10 pmol of each primer, 100 μM of each dNTP, 10 mM Tris-HCL pH 9.0, 20 mM (NH_4_)_2_SO_4_, 0.01% Tween 20, 1.5 mM MgCl_2 _and 0.3 Units Goldstar *Taq *DNA polymerase (Eurogentec, Maastricht, The Netherlands). The optimized PCR conditions used for the database construction were 94°C for 3 min. followed by 30 cycles of 94°C for 30 s, at the calculated annealing temperature for 30 s, 72°C for 60 s and a final extension at 72°C for 3 min. Unlabeled primers were obtained from Isogen (Maarssen, The Netherlands), fluorescently labelled (HEX, NED, 6-FAM) primers from Applied Biosystems (Warrington, United Kingdom). The amplification products were separated on a 6% acrylamide gel and visualized with silver staining according to Promega Silver sequence DNA sequencing system (Promega, Leiden, The Netherlands) as described [34]. Fluorescent amplification products were combined (see Table 1) and purified using Multiscreen 96-well Sephadex G50 filtration plates (Millipore). One μl of purified sample was mixed with 10 μl of formamide loading buffer containing a ROX-labelled internal lane standard. After denaturation at 95°C for 3 min, followed by quenching on ice, 1 μl samples were loaded in a capillary sequencer (3700 POP6, ABI) and run for 1.5 h. Fragment sizes were determined automatically using Genescan 1.1 (ABI). All genotypes were analyzed using Genotyper 3.5 NT (ABI).

### Data analysis

A selection of 12 microsatellite markers with high quality patterns (see Table 1) was used for the characterization of the varieties. Screening of varieties in a first round revealed all existing alleles for each marker and allowed selection of a set of varieties representing all the alleles. These varieties were included in each following run and used as a reference for allele determination. In this way for each marker the alleles were assigned a name (a, b, c, etc.) based on an exact match to the length of the corresponding allele present in the reference variety, rather than as a particular length in base pairs. Only the presence of alleles was scored and recorded as a presence/absence (1/0) matrix. As a consequence, both AAB and ABB genotypes, for example, are scored and entered in the database as AB. We call this the 'allelic phenotype' [7,8,24,35] after Becher et al. [36] to distinguish it from the genotype. An allelic phenotype is not the same as a genotype, as it only includes information on the presence of alleles, not on the allele frequency [26]. We report the number of alleles per locus, the effective number of alleles, and the number of allelic phenotypes. The effective number of alleles (n_e_) is estimated as 1/Σp_i_^2^, where p_i _is the frequency of the i^th ^allele in the variety examined. We prefer calculating the effective number of alleles to the expected heterozygosity (which is 1-Σp_i_^2^). These two measures have a non-linear relationship (*n*_E _= 1/(1-*H*_exp_)), and the effective number of alleles scales better when there are many alleles. More importantly, it is less affected by our dominant way of scoring alleles, and has a straightforward interpretation even across ploidy levels. On the basis of individual allele scores Jaccard distances were calculated. The Jaccard distance and the related Dice distance ignore absence-absence pairs, whose number may be inflated by the dominant scoring of a codominant marker. The varieties were clustered using neighbour-joining in NTSYSpc 2.1.

SpaGeDi 1.0b [37], which can handle plants of different ploidy levels, was used to calculate genetic differentiation (F_st_) among varieties on the basis of the presence of alleles. The magnitude of the error in allele frequencies caused by scoring only presence/absence and ignoring all presence of more than one copy in diploid and triploid varieties, was estimated for the diploid plants through a comparison with the results of an analysis of codominantly scored data.

For the codominantly scored diploid plants also Nei's heterozygosity, gene diversity, allelic richness, and F_is _values were calculated per variety, using SpaGeDi.

## Authors' contributions

JdR and BV conceived and designed the study; GDE and IE performed the experiments; MJMS, IE, JdR, and GDE analyzed the data; MJMS, GDE and BV wrote the paper. All authors read and approved the final manuscript.

## Supplementary Material

Additional file 1 A table reporting gene diversity, allelic richness and Fis values per marker and variety. Click here for file

Additional file 2 **A PCO plot of the sugar beet varieties based on pairwise genetic distances between sugar beet varieties calculated using dominant scoring of alleles.** Triploid varieties: open circles; diploid varieties: filled circles. Click here for file
