# Supplementary material for: Characterisation of sugar beet (Beta vulgaris L. ssp. vulgaris) varieties using microsatellite markers
Source: BMC Genet. 2010 May 18;11:41. doi: 10.1186/1471-2156-11-41 (PMC2890681; doi:10.1186/1471-2156-11-41)
Supplement: Additional file 2 — A PCO plot of the sugar beet varieties based on pairwise genetic distances between sugar beet varieties calculated using dominant scoring of alleles. Triploid varieties: open circles; diploid varieties: filled circles. [file 1471-2156-11-41-S2.PPT]

## Slide 1
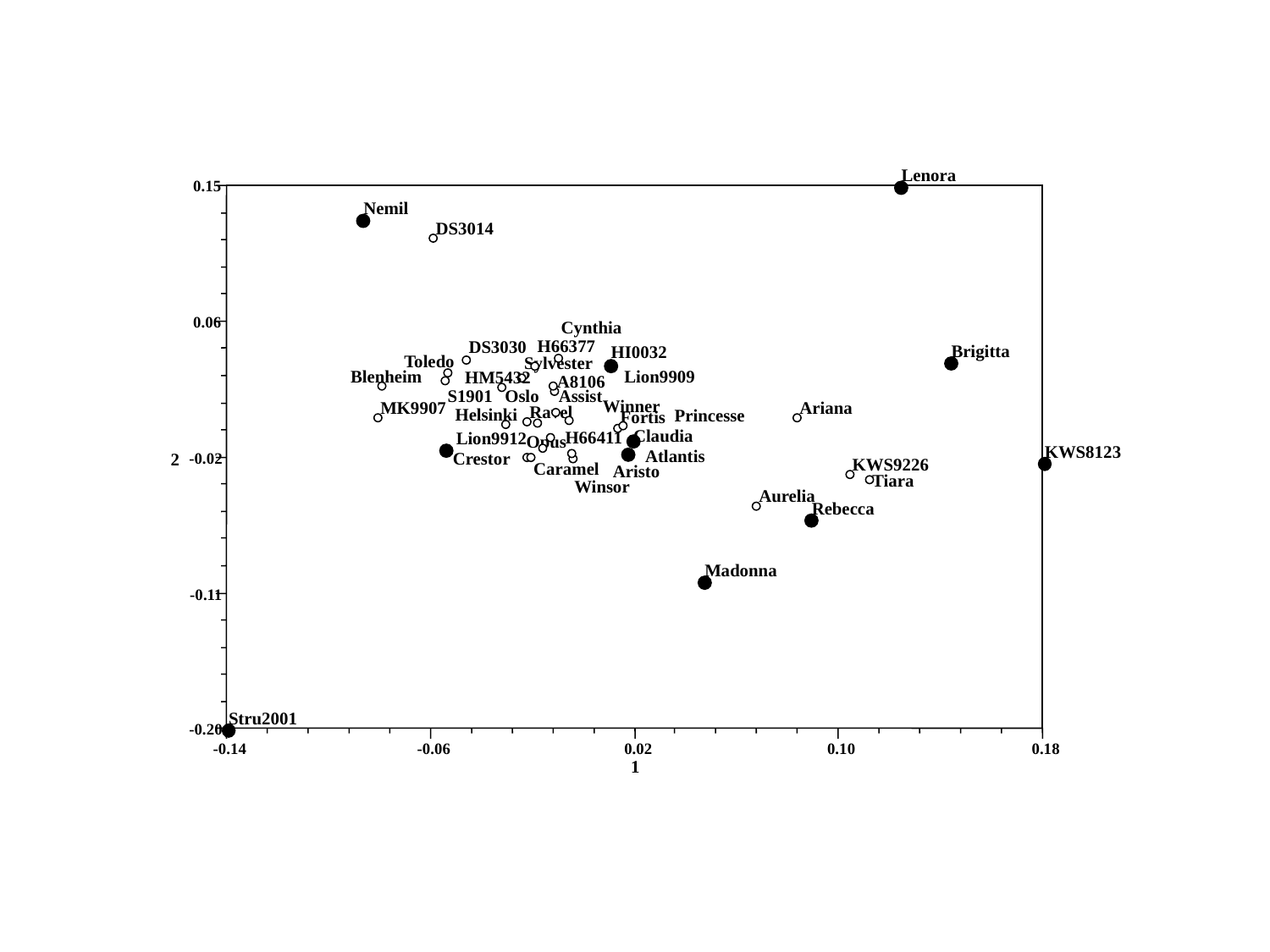

Lenora
0.15
Nemil
DS3014
0.06
Cynthia
H66377
DS3030
Brigitta
HI0032
Toledo
Sylvester
Blenheim
Lion9909
HM5432
A8106
Oslo
Assist
S1901
Winner
MK9907
Ariana
Ravel
Helsinki
Princesse
Fortis
Claudia
H66411
Lion9912
Opus
KWS8123
Atlantis
Crestor
2
-0.02
KWS9226
Caramel
Aristo
Tiara
Winsor
Aurelia
Rebecca
Madonna
-0.11
Stru2001
-0.20
-0.14
-0.06
0.02
0.10
0.18
1
